# Supplementary material for: MetaRibo-Seq measures translation in microbiomes
Source: Nat Commun. 2020 Jun 29;11:3268. doi: 10.1038/s41467-020-17081-z (PMC7324362; doi:10.1038/s41467-020-17081-z)
Supplement: Supplementary file 10 — Supplementary Data 7 [file 41467_2020_17081_MOESM10_ESM.zip › File2/Confidence_VeryHigh_Taxonomy/368591_out.krona.html]

Javascript must be enabled to view this page.

members
magnitude
magnitudeUnassigned
count
unassigned
taxon
rank

368591\_out

72

72
2
superkingdom

phylum
976
72

200643
72
class


SRS014459\_contig\_number\_contig-100\_542.231929SRS015065\_contig\_number\_contig-100\_605.218363SRS050974\_contig\_number\_contig-100\_2698.2699SRS1041039\_contig\_number\_17964SRS893380\_contig\_number\_1564
order
72
171549
5

171550
2
family

genus
239759
2

species

SRS075078\_contig\_number\_10555
1
1896968

1
28117
species

SRS050299\_contig\_number\_contig-100\_3574.75234

2005525
1
family

1
375288
genus

1
46503
species

SRS020508\_contig\_number\_1120

2005519
1
family

genus
397864
1

487174
1

SRS143895\_contig\_number\_31040
species

family
815
63

816
63
genus

1
29523
species

SRS019601\_contig\_number\_15706

species

SRS893279\_contig\_number\_15017
1
162156

2
820
species

SRS055966\_contig\_number\_176SRS077086\_contig\_number\_13551

19
1638779

SRS013800\_contig\_number\_15630SRS015782\_contig\_number\_9353SRS015960\_contig\_number\_779SRS016989\_contig\_number\_7815SRS018836\_contig\_number\_2952SRS019910\_contig\_number\_21373SRS020622\_contig\_number\_contig-100\_421.48976SRS043768\_contig\_number\_30681SRS043841\_contig\_number\_8872SRS044535\_contig\_number\_21701SRS050520\_contig\_number\_1056SRS052027\_contig\_number\_2839SRS056259\_contig\_number\_28458SRS064557\_contig\_number\_17054SRS077231\_contig\_number\_18407SRS077335\_contig\_number\_8014SRS100907\_contig\_number\_7645SRS1041090\_contig\_number\_2343SRS144506\_contig\_number\_38421
species

species

SRS018817\_contig\_number\_10723
310297
1

1
818

SRS143417\_contig\_number\_2248
species

species

SRS075878\_contig\_number\_17601SRS098881\_contig\_number\_13408
2
817

species

SRS014979\_contig\_number\_35181SRS015190\_contig\_number\_14835SRS015264\_contig\_number\_10299SRS015431\_contig\_number\_18426SRS016495\_contig\_number\_19834SRS016897\_contig\_number\_contig-100\_63.21632SRS016954\_contig\_number\_26301SRS017821\_contig\_number\_12524SRS018351\_contig\_number\_16438SRS018888\_contig\_number\_7208SRS019068\_contig\_number\_39879SRS019808\_contig\_number\_18040SRS019968\_contig\_number\_10714SRS020394\_contig\_number\_16661SRS021948\_contig\_number\_22225SRS024625\_contig\_number\_21237SRS043411\_contig\_number\_13496SRS046369\_contig\_number\_11613SRS052576\_contig\_number\_contig-100\_25.15534SRS053214\_contig\_number\_13302SRS056273\_contig\_number\_10108SRS063127\_contig\_number\_2643SRS065504\_contig\_number\_11127SRS075773\_contig\_number\_contig-100\_2359.205095SRS077231\_contig\_number\_5530SRS077730\_contig\_number\_13092SRS103987\_contig\_number\_33460SRS1041036\_contig\_number\_4088SRS1041090\_contig\_number\_455SRS1041144\_contig\_number\_8210SRS104912\_contig\_number\_3602SRS1055049\_contig\_number\_1054SRS144183\_contig\_number\_contig-100\_185.214062SRS145308\_contig\_number\_2602SRS893382\_contig\_number\_4595SRS893383\_contig\_number\_29163
36
821
